# Evaluation of the Medicinal Herb *Graptopetalum paraguayense* as a Treatment for Liver Cancer

**DOI:** 10.1371/journal.pone.0121298

**Published:** 2015-04-07

**Authors:** Wei-Hsiang Hsu, Chia-Chuan Chang, Kai-Wen Huang, Yi-Chen Chen, Shih-Lan Hsu, Li-Chen Wu, Ann-Ping Tsou, Jin-Mei Lai, Chi-Ying F. Huang

**Affiliations:** 1 Institute of Biopharmaceutical Sciences, National Yang-Ming University, Taipei, Taiwan; 2 School of Pharmacy, National Taiwan University, Taipei, Taiwan; 3 Department of Surgery and Hepatitis Research Center, National Taiwan University Hospital, National Taiwan University, Taipei, Taiwan; 4 Department of Medical Research, Taichung Veterans General Hospital, Taichung, Taiwan; 5 Department of Applied Chemistry and Graduate Institute of Biomedicine and Biomedical Technology, National Chi Nan University, Puli, Nantou, Taiwan; 6 Department of Biotechnology and Laboratory Science in Medicine, National Yang-Ming University, Taipei, Taiwan; 7 Department of Life Science, Fu-Jen Catholic University, New Taipei City, Taiwan; Taipei Medical University, TAIWAN

## Abstract

**Background:**

Hepatocellular carcinoma (HCC) is the fifth most common malignancy and the third most common cause of cancer-related death worldwide. Sorafenib is the only drug for patients with advanced-stage hepatocellular carcinoma (HCC) that has been shown to confer a survival benefit to patients with HCC; however, it has many side effects. Thus, alternate therapeutic strategies with improved safety and therapeutic efficacy for the management of HCC should be developed.

**Methods and Findings:**

We demonstrate that an extract of *Graptopetalum paraguayense* (GP) down-regulated the expression levels of several onco-proteins, including AURKA, AURKB, and FLJ10540, in HCC cells. To isolate the active components in the GP extracts, we prepared extracts fractions and assessed their effects on the expression of onco-proteins in HCC cells. The fraction designated HH-F3 was enriched in active ingredients, exhibited cytotoxic effects, and suppressed the expression of the onco-proteins in HCC cells. The structure of the main active compound in HH-F3 was found to be similar to that of the proanthocyanidin compounds derived from *Rhodiola rosea*. In addition, a distinct new compound rich in 3, 4, 5-trihydroxy benzylic moieties was identified in the HH-F3 preparations. Mechanistic studies indicated that HH-F3 induced apoptosis in HCC cells by promoting the loss of mitochondrial membrane potential and the production of reactive oxygen species. HH-F3 also enhanced PTEN expression and decreased AKT phosphorylation at Ser473 in a concentration-dependent manner in HCC cells. Moreover combination of GP or HH-F3 and sorafenib synergistically inhibits the proliferation of Huh7 cells. The treatment of a rat model with diethylnitrosamine (DEN)-induced liver cancer with extracts of GP and HH-F3 decreased hepatic collagen contents and inhibited tumor growth.

**Conclusions:**

These results indicate that GP extracts and HH-F3 can protect the liver by suppressing tumor growth; consequently, these compounds could be considered for the treatment of HCC.

## Introduction

Alcoholism, viral hepatitis and nonalcoholic steatohepatitis are the three major causes of chronic liver inflammation and injury. Chronic liver inflammation can lead to liver fibrosis, cirrhosis and hepatocellular carcinoma (HCC), which is the most common form of liver cancer and accounts for 70% to 85% of primary hepatic malignancies in adults. More than 80% of patients with HCC are diagnosed at an advanced stage of disease at which surgical treatment is no longer indicated. In general, patients with unresectable HCC have a poor prognosis and rarely benefit from nonsurgical treatments such as systematic chemotherapy or chemoembolization [1, 2].

Abnormal signaling in the PI3K/PTEN/AKT pathway contributes to the development of a variety of hepatic diseases, including non-alcoholic fatty liver disease (NAFLD), non-alcoholic steatohepatitis (NASH), alcoholic liver disease (ALD), viral hepatitis and HCC [3]. The PI3K/PTEN/AKT pathway is activated by the epigenetic suppression of PTEN and/or mutations in or amplification of individual components of the pathway in approximately 40–50% of patients with HCC [4–6]. Experimentally, the deletion of PTEN or the overexpression of AKT in the livers of mice results in steatosis and tumor development [7, 8]. These experimental observations strongly suggest that the PI3K/PTEN/AKT pathway plays an important role in hepatotumorigenesis.

Sorafenib, a multi-target kinase inhibitor that was approved for the treatment of HCC in 2007 [9], is the only standard chemotherapeutic drug for patients with advanced-stage HCC [10]. This drug has been shown to inhibit tumor cell proliferation by blocking the Ras/Raf/MAPK pathway and angiogenesis by blocking both VEGFR and PDGFR signaling, thus slowing the growth of new blood vessels within the tumor [11]. In a double-blind, randomized, controlled clinical trial (RCT) with a primary endpoint of overall survival [12], sorafenib increased the survival time of HCC patients from 7.9 to 10.7 months [13]. Sorafenib is the only drug that has been shown to confer a survival benefit to patients with HCC; however, it has many side effects. Currently, treatment options for patients with advanced HCC are limited. Thus, alternate therapeutic strategies with improved safety and therapeutic efficacy for the management of HCC should be developed.

Herbal medicines have been used for thousands of years to treat a wide range of diseases, including inflammatory diseases and chronic liver diseases (including hepatitis, hepatic fibrosis and HCC). In this study, we focused on *Graptopetalum paraguayense* (GP), a traditional Chinese herb that possesses several health benefits. According to its archaic Chinese prescription, GP is able to alleviate hepatic disorders, lower blood pressure, whiten skin, relieve pain, treat infections, inhibit inflammation and improve brain function [14–16]. *In vitro* studies have shown that extracts from the leaves of GP inhibit tyrosinase and angiotensin-converting enzyme and scavenge free radicals [14–16]. Extracts from the stem of GP cultured with cells from the human HCC HepG2 cell line have also been shown to exhibit antioxidant and anti-proliferative properties [17]. Water-based extracts of GP have also been shown to have antioxidative and anti-inflammatory properties that protect cells from CCl_4_-induced oxidative liver damage [18]. Data from our previous microarray profiling study showed that the expression of various genes related to metabolism, cell growth and/or maintenance was restored to near-normal levels in DMN-treated rats treated with GP [19]. Our previous study also showed that the administration of GP mitigated chemical-induced hepatic damage and fibrosis *in vivo* and suppressed hepatic stellate cell (HSC) and Kupffer cell activation *in vitro*.

The abovementioned findings suggest that GP may represent a therapeutic option for treating hepatic inflammation and fibrosis [20]. In this study, we demonstrate that GP and its HH-F3 fraction have anti-cancer effects in the rat model of diethylnitrosamine (DEN)-induced liver cancer. We subsequently show that extracts of GP and HH-F3 induce apoptotic cell death in HCC cells, suggesting that GP and HH-F3 could be used for the prevention or treatment of HCC.

## Materials and Methods

### Cell lines

Huh7 and PLC5 cell lines were obtained from Dr. Zhong-Zhe Lin, National Taiwan University Hospital, Taiwan. HepG2 cell line was purchased from American Type Culture Collection (ATCC, http://www.atcc.org/en.aspx). Mahlavu cell lines were provided by Dr. Muh-Hwa Yang, Institute of Clinical Medicine, National Yang-Ming University, Taiwan. Human hepatocyte was purchased from ScienCell Research Laboratories (http://www.sciencellonline.com/).

### GP and *Rhodiola rosea* extraction, purification and characterization

GP leaves were ground and lyophilized into a powder at -20°C and stored in a moisture buster at 25°C before extraction. First, 1.5 g of GP powder was vortexed with 10 ml of 100% methanol (MeOH) for 5 min and centrifuged at 1,500 *g* for 5 min. The supernatant was removed and various extracts were prepared by re-suspending the pellets in 10 ml of H_2_O, 100% acetone, 100% methanol, 100% ethanol, 70% ethanol, 50% ethanol, 100% DMSO or 30% DMSO. The suspension was vortexed for 5 min, centrifuged twice at 1,500 *g* for 5 min, centrifuged again at 9,300 *g* for 5 min and filtered through a 0.45-μm filter in a laminar flow hood at room temperature. The 30% DMSO supernatant was either stored at -20°C as a 150 mg/ml stock solution (referred to as 30% DMSO GP extracts) or fractionated into four fractions (F1–F4) using a Sephadex LH-20 (GE Healthcare Bio-Sciences AB, Uppsala, Sweden) column (Method I). To simplify the preparation protocols, direct dialysis of the 30% DMSO GP extracts against water was also undertaken (Method II). Colorimetric assays on the 30% DMSO GP extracts for identification of hydrolyzable and condensed tannins were performed [21]. The HCC cells were treated with the extracts, and AURKA, AURKB and FLJ10540 protein levels were analyzed by Western blot; active molecules in the F3 fraction (referred to as the HH-F3 fraction) were identified. The HH-F3 fraction was further analyzed by HPLC with a UV detector (Shimadzu SPD-M10A), a normal-phase HPLC column (Phenomenex Luna 5u Silica (2) 100A, 4.6 × 250 mm) and ^1^H- and ^13^C-NMR spectra to identify the structure of the active molecules. GP extracts and the HH-F3 fraction were also subjected to dialysis against water using a dialysis membrane (MWCO 12–14,000) (Spectrum Laboratories, Rancho Dominguez, CA) to obtain active compounds. *Rhodiola rosea* plants were lyophilized into a powder and stored in a moisture buster at 25°C prior to extraction. A 1.5 g sample of *Rhodiola rosea* powder was dissolved in 10 ml of H_2_O, centrifuged at 1,500 *g* for 5 min and filtered through a 0.45-μm filter in the laminar flow hood at room temperature. The samples were stored at -20°C as 150 mg/ml stock solutions.

### Western blot

The lysates of HCC cells were subjected to SDS-PAGE to resolve the expressed proteins and transferred to polyvinylidene difluoride (PVDF) membranes using a Bio-Rad transfer system. After transfer, the membranes were stained with Ponceau S to confirm the efficiency and uniformity of the protein transfer. The membranes were blocked with 5% non-fat skim milk at room temperature for 30 min and incubated with the primary antibody at 4°C overnight. Subsequently, the membranes were washed three times (10 min each) with 1x Tris-buffered saline containing Tween-20 (TBST) and incubated with HRP-conjugated secondary antibodies for 2 hrs. HRP substrate peroxide solution/luminol reagents (Immobilon Western Chemiluminescent Substrate, Millipore; mixed at a 1:1 ratio) were added and the secondary antibody signals on the membranes were visualized with a Fujifilm LAS4000 luminescent image analysis system. The following primary antibodies were used: anti-PTEN, anti-AKT, anti-P70S6K, anti-caspase 3 and anti-PARP (Cell Signaling); anti-BCL2, anti-BCL-XL and anti-caspase 9 (GeneTex); anti-AURKA and anti-AURKB (BD Biosciences); and anti-FLJ10540 (Abnova). All antibodies were used at a dilution of 1:1,000.

### Viability assay

The cells were seeded in 24-well plates (4,000–5,000 cells/well) for overnight and then treated with the 30% DMSO GP extracts or the HH-F3 fraction for 0, 24, 48, or 72 hours. After treatment, the cells were gently washed 3 times with 1x PBS (137 mM NaCl, 2.7 mM KCl, 10 mM Na_2_HPO_4_, 2 mM KH_2_PO_4_) and then incubated with 0.5 μg/ml 3-(4,5-cimethylthiazol-2-yl)-2,5-diphenyl tetrazolium bromide (MTT, Sigma-Aldrich) for 2 hours. The medium was removed, and the deep-blue crystals were dissolved with 100% DMSO at room temperature for 10 minutes. OD values were measured at 570 nm with an ELISA reader.

### Cell counting

The cells were seeded in 12-well plates (10,000–20,000 cells/well) and incubated overnight. The next day, the cells were treated with the HH-F3 fraction for 0, 24, 48 or 72 hrs. The cells were then trypsinized, treated with 0.4% trypan blue and counted.

### Cell cycle analysis and flow cytometry

After the cells had been trypsinized and washed three times with PBS, they were centrifuged at 800 *g* for 5 min. The cells were then resuspended in 70% ethanol in PBS and kept at -20°C for more than 16 hrs. The cells were centrifuged at 800 *g* for 5 min, and the cell pellets were resuspended in cold PBS containing 100 μg/ml RNAse A (Sigma-Aldrich) for 20 min. The cells were then stained with 20 μg/ml propidium iodide (PI, Sigma-Aldrich) for 20–30 min, and the DNA content was measured and analyzed using a BD FACSCanto and Flow Jo analysis software, respectively.

### Mitochondrial membrane potential assay

The mitochondrial membrane potential was analyzed using 5, 5', 6, 6'-tetrachloro-l, 1', 3, 3'-tetraethylbenzimidazolcarbocyanine iodide (JC-1) purchased from Cayman Chemical Co. The cultured cells were seeded in 96-well black plates at a density of 7,000 cells/well, incubated overnight and treated with or without the HH-F3 fraction for 48 hrs. The JC-1 staining solution was added to each well and mixed gently at 37°C for 15–30 min in the dark. The plates were centrifuged at 400 *g* at room temperature for 5 min and the supernatant was removed from each well. The JC-1 assay buffer was added to each well, the plates were centrifuged for 5 min at 400 *g* at room temperature and the supernatant was removed from each well. Finally, JC-1 assay buffer was added to each well, and the plates were analyzed using a fluorescent plate reader.

### Measurement of ROS levels

The intracellular generation of superoxide radicals (O_2_^-^) was assessed by hydroethidine fluorescence (AAT Bioquest, Inc.). The cells were treated with or without the HH-F3 fraction for 48 hrs. Hydroethidine (10 μM) was added to each well and mixed gently for 30–60 min at 37°C in the dark. Cellular fluorescence was monitored at an excitation wavelength of 520 nm and an emission wavelength of 610 nm. Intracellular peroxide levels were measured using dichlorofluorescein (DCFH) diacetate (Marker Gene Technologies, Inc.). After the cells had been treated with the HH-F3 fraction for 48 hrs, the medium was aspirated and the cells were washed twice with PBS. The cells were then incubated with DCFH at a final concentration of 20 μM in serum-free medium for 30–60 min at 37°C in the dark, washed again with PBS and maintained in 200 μl of culture medium. Cellular fluorescence was monitored at an excitation wavelength of 485 nm and an emission wavelength of 528 nm.

### Animals, the experimental environment, and protocol

The Animal Care and Use committee of the College of Medicine, National Taiwan University, approved all experimental protocols. All experiments were conducted according to the animal care guidelines of the university.

One hundred and twenty six-week-old male Wistar albino rats (150–180 g) were used. All animals were allowed to acclimate for seven days and provided standard chow and water *ad libitum* during the experimental period.

The rats were randomly assigned to the normal group (N = 10), the diethylnitrosamine (DEN) group (N = 30), the low-dose GP group (N = 30) or the high-dose GP group (N = 30). Five additional rats were included in the HH-F3-treated group. For all groups except the normal group, the sole source of drinking water for 63 days was an aqueous solution of 100 ppm (v/v) DEN (Sigma-Aldrich, St. Louis, MO, USA) that was changed daily; starting on day 64, the rats were provided with tap water for another 14 days. The DEN solution was prepared each week and consisted of an individualized dose calculated according to the weight gain/loss experienced by the animal in response to the previous dose. Liver tumors were observed starting on day 42, and liver fibrosis was observed starting on day 63. During the experimental period, the animals were weighed weekly to calculate weight gain; in addition, the amount of water consumed by the rats was measured every week. The rats in the low-dose group received 0.6 g/rat of lyophilized GP powder; the rats in the high-dose group received 1.8 g/rat of lyophilized GP powder; the rats in the HH-F3 group received 0.036 g/rat of lyophilized HH-F3 powder every day for three weeks beginning on day 42.

### Harvesting procedure and morphologic evaluation of the liver

All animals were euthanized on day 84. The animals were fasted overnight and sacrificed by CO_2_ inhalation. After the rats had been sacrificed, their bodies, livers and spleens were weighed; a midline laparotomy was performed and the conditions of the organs were noted at necropsy. All lobes of the liver were promptly harvested and thoroughly examined to describe the surface of the liver and to characterize the development of liver foci, persistent nodules (PNs) or cancer at every time point. Subsequently, the liver was cut into 5-mm sections. All macroscopically visible nodules on the liver surface and in the 5-mm sections were counted and measured.

### Tumor burden assessment

To establish the course of tumor development in the animals exposed to DEN, all lobes of the liver were promptly harvested, and all macroscopically visible nodules on the liver surface and in the 5-mm sections were counted and measured. Liver tumor burdens were determined by estimating the sums of the volumes of all tumor nodules greater than 3 mm in diameter for each animal; the tumor burdens were then compared among the groups.

### Bile flow rate

Prior to sacrifice, the animals were anesthetized with 80 mg/kg of ketamine and their bile flow rate was measured with a PE10 silicon tube placed in the common bile duct and connected to a calculated polyethylene tube. Bile flow into the tube was measured at 5-min intervals.

### Histopathological evaluation

After the blood had been drained, 5-mm-thick tissue slices containing tumors were dissected from each lobe of the liver. Sections (5 μm) were cut and stained with hematoxylin and eosin for histopathological analysis using published diagnostic criteria.

### Immunohistochemical staining for α-smooth muscle actin (α-SMA)

The liver samples were fixed with formalin, embedded in paraffin and sectioned into 5-μm sections. The sections were deparaffinized, rehydrated and treated with 0.03% hydrogen peroxide for 10 min to quench endogenous peroxidase activity. Following two washes with PBS, the sections were incubated for 1 hr at room temperature with a mouse anti-human α-SMA monoclonal antibody (1:50 dilution, Dako Cytomation, Denmark). For α-SMA staining, the sections were washed and incubated with a secondary rabbit anti-mouse IgG antibody (1:200 dilution) at room temperature for 1 hr. The sections were then developed similarly. After staining, the sections were counterstained with hematoxylin for microscopy analyses. The percentage of α-SMA-positive areas (mm^2^/cm^2^ of liver section) was determined using an HC-2500 digital camera system (Fuji Photo Film), Adobe Photoshop version 5.0J and Image-Pro Plus version 3.0.1J.

### Assay for hydroxyproline content in the liver

Liver specimens were weighed, and 20 mg of the frozen samples was hydrolyzed in 20 ml of 6 N HCl and carefully ground; 6 N HCl was then added to obtain a total volume of 30 ml/mg of tissue. The ground tissue was hydrolyzed at 120°C for 16 hrs. After a brief period of cooling on ice and centrifugation at 8,000 *g* for 10 min, the supernatant was removed and placed in a new tube; the volume lost to evaporation was replenished with water. An equal volume of 6 N NaOH was added and mixed, and the pH of the solution was adjusted to pH 4–9 using litmus paper. Forty microliters of the neutralized sample solution was added to the wells of a 96-well ELISA plate and oxidized using a solution containing 5 ml of 7% chloramine T (Sigma-Aldrich) and 20 ml of acetate/citrate buffer. One hundred and fifty milliliters of Ehrlich’s solution was added. The final mixture was incubated at 60°C for 35 min and at room temperature for 10 min; the absorbance was then determined at 560 nm. Standard solutions containing 100, 80, 60, 40, 20 and 0 mg/ml of authentic 4-hydroxy-L-proline (Sigma-Aldrich) were treated in a similar manner. The standard curve was linear in this range of concentrations (*r* = 0.99). The hepatic hydroxyproline level was expressed as mg of hydroxyproline/g of wet liver weight. All assays were performed in triplicate.

### Statistical analysis

All results were expressed as the mean ± standard error of the mean. Levels of significance were evaluated by two-tailed paired Student’s *t*-test or one-way analysis of variance (ANOVA). *P* < 0.05 was considered statistically significant.

## Results

### The anti-proliferative effects of different GP preparations on Huh7 and Mahlavu cells

**Fig 1:**
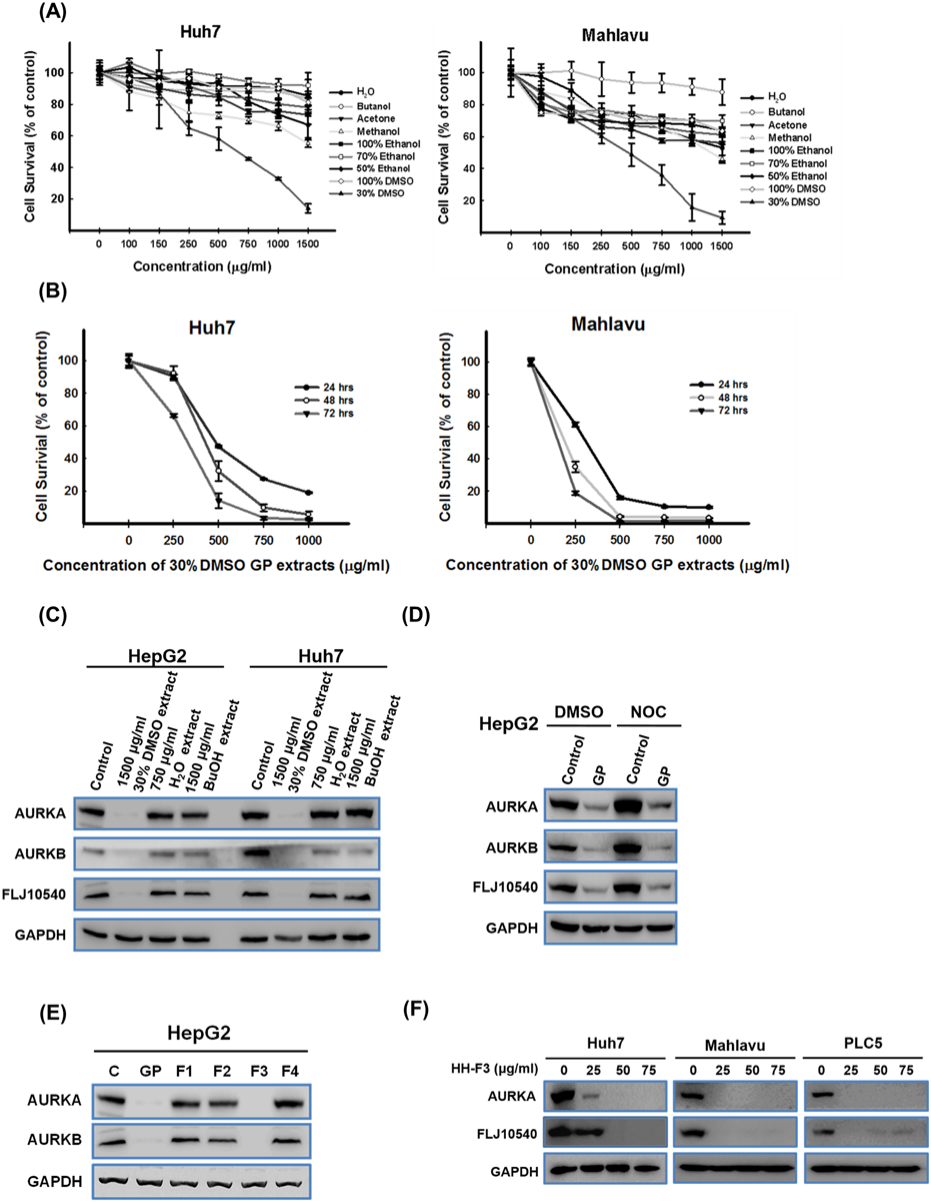
GP extracts and their fractions regulate oncogenic protein expression in HCC cell lines. Huh7 and Mahlavu cells were treated with GP extracts prepared in water, butanol, acetone, methanol, 100% ethanol, 70% ethanol, 50% ethanol, 100% DMSO, or 30% DMSO at concentrations of 100, 150, 250, 500, 750, 1000 and 1500 μg/ml for 72 hrs. The viability of the treated cells was determined using MTT assays. The 30% DMSO GP extracts was associated with the greatest inhibition of growth of Huh7 and Mahlavu cells after 72 hrs. (B) Huh7 and Mahlavu cells were treated with concentrations of 0, 250, 500, 750 and 1000 μg/ml of the 30% DMSO GP extracts for 24, 48, and 72 hrs, and MTT assays were conducted. The IC_50_ concentrations of the 30% DMSO GP extracts for growth inhibition of the Huh7 and Mahlavu cells were approximately 500 and 250 μg/ml, respectively, after 72 hrs of treatment. (C) HepG2 and Huh7 cells were treated with the GP extracts prepared in 30% DMSO, water or butanol (BuOH) for 48 hrs. The levels of AURKA, AURKB and FLJ10540 were measured by Western blot analysis. The expression levels of AURKA, AURKB and FLJ10540 in HepG2 and Huh7 cells were suppressed by the 30% DMSO GP extracts but not by the fractions prepared in water or butanol. (D) HepG2 cells were treated with 75 ng/ml of the synchronizing agent nocodazole (NOC) for 18 hrs; the cells were then treated with 750 μg/ml of the 30% DMSO GP extracts or vehicle control (30% DMSO) for another 3 hrs. Western blotting was performed using anti-FLJ10540, anti-AURKA and anti-AURKB antibodies. (E) HepG2 cells were treated with the 30% DMSO GP extracts and its fractions (HH-F1, HH-F2, HH-F3 and HH-F4) for 3 hrs. AURKA and AURKB expression levels in HepG2 cells were suppressed by the HH-F3 fraction but not by the other fractions. (F) Huh7, Mahlavu and PLC5 cells were treated with various concentrations of HH-F3 for 48 hrs. The expression of AURKA and FLJ10540 proteins was assessed by immunoblot analysis. HH-F3 reduced AURKA and FLJ10540 protein levels in a concentration-dependent manner.

To test the potential biological effects of GP, various GP extracts were prepared using water, butanol, acetone, methanol, 100% ethanol, 70% ethanol, 50% ethanol, 100% DMSO or 30% DMSO and used to treat human HCC cells. The growth inhibition induced by the different GP extracts was examined. Data from the MTT assays indicated that the 30% DMSO extracts significantly reduced the viability of Huh7 and Mahlavu cells (Fig 1A); more specifically, concentrations of 500 and 250 μg/ml resulted in 50% inhibition of cell viability (IC_50_) 72 hrs post-treatment for Huh7 and Mahlavu cells, respectively (Fig 1B).

### GP reduces AURKA, AURKB and FLJ10540 protein expression levels during both interphase and mitosis in activated hepatic stellate cells and HCC cell lines

AURKA, AURKB and FLJ10540 are oncogenes that are commonly overexpressed in HCC [22–24]. Accidentally, we found that the 30% DMSO GP extracts inhibited the protein expression levels of FLJ10540, AURKA and AURKB in HCC cell lines (HepG2 and Huh7) in a concentration-dependent manner (Fig 1C, S1 Fig). In contrast, the GP extracts prepared in either water or butanol did not inhibit the protein expression levels of AURKA or AURKB in HepG2 cells after 72 hrs of treatment (Fig 1C). It is well known that the expression levels of AURKA, AURKB and FLJ10540 are higher during metaphase than during interphase [25–27]. We therefore investigated whether the 30% DMSO GP extracts could inhibit the expression of these proteins during mitosis in HCC cells. HepG2 cells were first treated with 75 ng/ml of nocodazole for 18 hrs; these cells were then treated with the 30% DMSO GP extracts for 3 hrs (without washing out the nocodazole). AURKA, AURKB and FLJ10540 expression levels were high during mitosis. In cells treated with the 30% DMSO GP extracts, the protein expression levels of AURKA, AURKB and FLJ10540 were decreased during interphase and metaphase (Fig 1D); in contrast, no significant changes in the expression levels of other mitotic proteins (PIN1, HURP and PLK) were observed (data not shown).

### HH-F3 suppresses AURKA protein expression in HCC cell lines

Next, using a Sephadex LH-20 column, we obtained four fractions from the 30% DMSO GP extracts (S2A Fig). Western blot analysis conducted 3 hrs post-treatment showed that only the third fraction (HH-F3) suppressed the expression of AURKA and AURKB in HepG2 cells (Fig 1E). Next, Huh7, Mahlavu and PLC5 cells were treated without or with 25, 50 and 75 μg/ml of the HH-F3 fraction for 48 hrs. The expression of AURKA and FLJ10540 in all three HCC cell lines was significantly suppressed by HH-F3 (Fig 1F). To investigate whether the inhibitory effects of HH-F3 occurred at the transcriptional level, we examined the variation in gene expression levels of *FLJ10540* and *AURKA*, *AURKB* and *AURKC* (members of the Aurora kinase family of genes). HepG2 cells were treated with 50 μg/ml of the HH-F3 fraction for 6 hrs, and gene expression and protein levels were measured by microarray (U133A chip, Affymetrix) and Western blot, respectively. There was almost no change in the gene expression levels of any of the abovementioned genes after treatment with HH-F3; however, decreases in protein levels were observed (data not shown). These results suggest that the HH-F3 fraction regulated the expression of FLJ10540 and the Aurora kinase family molecules at the translational level rather than at the transcriptional level.

### Identification of the active components in GP

**Fig 2:**
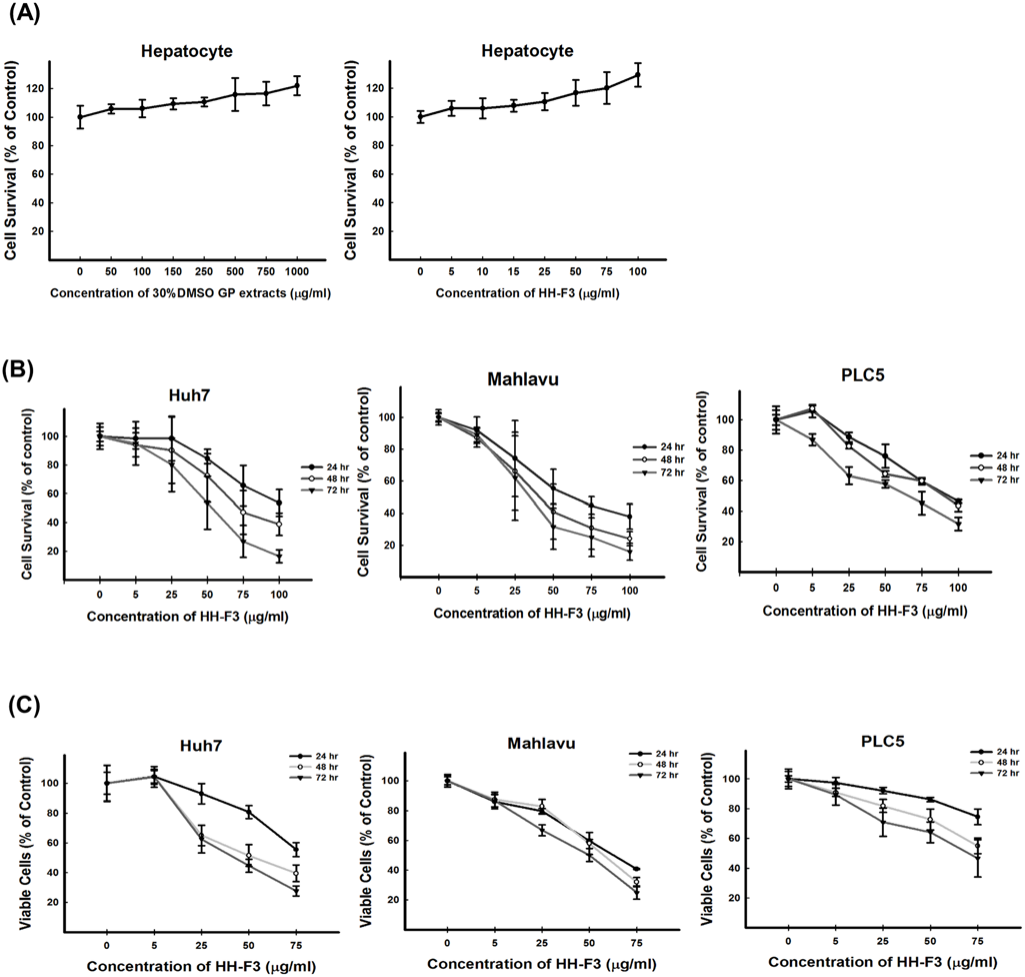
The HH-F3 fraction inhibits the growth of HCC cells. (A) Human hepatocyte were treated with various concentrations of 30% DMSO GP extracts/HH-F3 for 72 hrs. Data are expressed as the mean ± standard deviation (SD) of three independent experiments. Huh7, Mahlavu and PLC5 cells were treated with 5, 25, 50 and 75 μg/ml of the HH-F3 fraction at for 24, 48 and 72 hrs then subjected to the MTT assay (B) and trypan blue assay (C). The IC_50_ concentrations of HH-F3 for the growth inhibition of Huh7, Mahlavu and PLC5 cells were approximately 50, 37.5 and 75 μg/ml, respectively, after 72 hrs of treatment.

To simplify the preparation protocols, another method (Method II) by direct dialysis of the 30% DMSO GP extracts against water was used. The physiochemical properties of the compounds obtained using Method II are listed in S1 Table, and these data indicated that the fractions obtained with Method I (the method initially used to prepare HH-F3) and Method II were identical. After lyophilization, characteristic pink color and partial silver metal-like luster of the HH-F3 fraction was observed. According to the broadened aromatic signals in ^1^H and ^13^C NMR spectra, the major components of the HH-F3 fraction were identified as polyphenolic compounds; more specifically, tannins. A colorimetric assay (OD_500_) for the quantification of the condensed tannin using catechin as the standard showed that the total tannin content of the HH-F3 fraction was approximately 68% (data not shown). The HPLC fingerprint of the HH-F3 fraction (S2 Fig 2B) revealed that this fraction contained two groups of compounds (Groups A and B) with distinct ranges of molecular weights; Group B contained one major and one minor component. These findings revealed the the HH-F3 fraction was rich in condensed tannins and contained compounds with high molecular weights.

Because no polymeric compounds have yet been isolated from GP, the polyphenolic compounds from *Rhodiola rosea* (golden root), another herb of the Crassulaceae family, were used as reference compounds for the structural identification of compounds in the HH-F3 fraction. *R*. *rosea* has been reported to contain condensed tannins (CTs). The chemical properties of the main compounds in the HH-F3a sub-fraction, both from Methods I and II, were very similar to those of the CTs in *R*. *rosea* (golden root). In addition, because CT compounds are frequently found in many common grape species, *Vitis vinifera* CTs were also used as reference compounds. S1 Table summaries the physiochemical properties of the polymeric proanthocyanidin from *R*. *rosea* and *Vitis vinifera*. Its high ratio in 3, 4, 5-trihydroxy benzylic substituents of HH-F3a (identified by the ^13^C chemical shifts at 105 ppm and 109 ppm for C-2’/6’ and C-2”/6” of the EGCG unit, respectively; spectra not shown) was very similar to that of the compound found in *R*. *rosea*, but much higher than the compound found in grape skins and seeds (see S1 Table). Accordingly, the CT presented in HH-F3a was proposed as a polymeric proanthocyanidin with a dominative prodelphinidin repeating unit and (4→8)-linkages (see S2C Fig). Chemical characterization of HH-F3a and structural elucidation of the repeating unit after chemical degradation by thiolysis will be published separately.

### The HH-F3 fraction reduces the viability of HCC cells

To investigate the effects of the HH-F3 fraction on cell viability, primary hepatocyte, Huh7, Mahlavu and PLC5 cells were treated with HH-F3 at concentrations of 0, 5, 25, 50, 75 and 100 μg/ml for 24, 48 and 72 hrs. The MTT assay showed that both 30% DMSO GP extracts and HH-F3 treatment did not inhibit the survival of human hepatocytes (Fig 2A). The IC_50_ concentrations of the HH-F3 fraction for the Huh7, Mahlavu and PLC5 cells at 72 hrs were 50, 37.5 and 75 μg/ml, respectively (Fig 2B). To further confirm this finding, cell survival was assessed by trypan blue staining of the Huh7, Mahlavu and PLC5 cells. The HH-F3 fraction reduced cell survival in a concentration-dependent manner (Fig 2C). Finally, both 30% DMSO GP extracts and HH-F3 fraction seemed to inhibit the viability of the HCC cell lines better than other cancer cell lines test (S3 Table).

### The HH-F3 fraction decreases mitochondrial membrane potential and increases the production of reactive oxygen species in HCC cell lines

**Fig 3:**
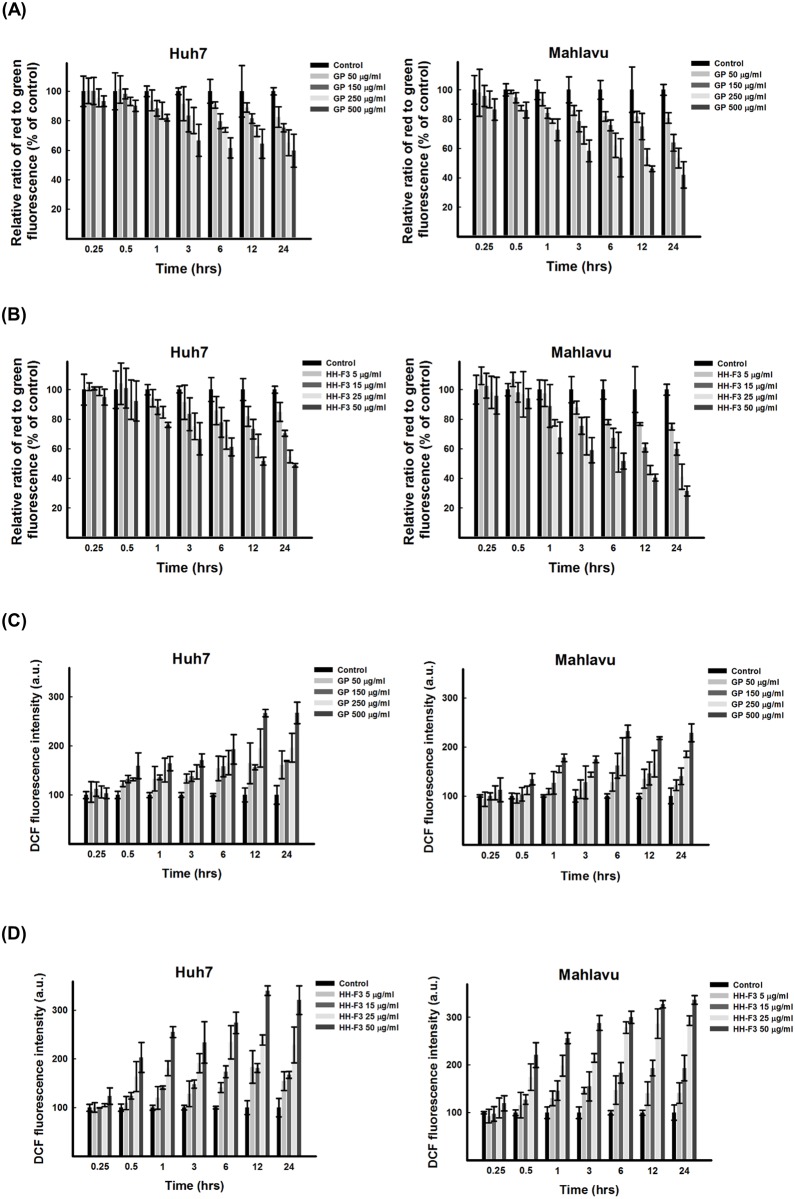
Disruption of mitochondrial membrane potential and increase ROS generation in HCC cells treated with 30% DMSO GP extracts and HH-F3. (A-B) The mitochondrial membrane potential (ΔΨ) in Huh7 and Mahlavu cells was analyzed using the JC-1 mitochondrial membrane potential assay. The ΔΨ of the cells was lower in the HCC cells treated with the 30% DMSO GP extracts and HH-F3 than in the control HCC cells. The treatment effect as a function of time (0.25, 0.5, 1, 3, 6, 12, and 24 hrs) is shown (n = 3). (C-D) Intracellular peroxide levels, as measured by DCFH fluorescence, were increased after treatment with different concentrations of 30% DMSO GP extracts and HH-F3. The treatment effect as a function of time (0.25, 0.5, 1, 3, 6, 12, and 24 hrs) is shown (n = 3).

We tested whether the mitochondrial membrane potential, a marker of the activation of the intrinsic apoptotic pathway, was altered in HCC cells treated with HH-F3. Huh7 and Mahlavu cells were treated with 5, 25 and 50 μg/ml of the HH-F3 fraction, and the mitochondrial membrane potential of the cells was examined after 0.25, 0.5, 1, 3, 6, 12, 24 and 48 hrs of treatment. The number of apoptotic cells was higher in the treated groups than in the control groups. This effect was coincident with mitochondrial membrane potential (ΔΨ) dysfunction, indicating that mitochondrial membrane potentials were decreased in Huh7 and Mahlavu cells treated with HH-F3 (Fig 3A and 3B, S3A Fig).

**Fig 4:**
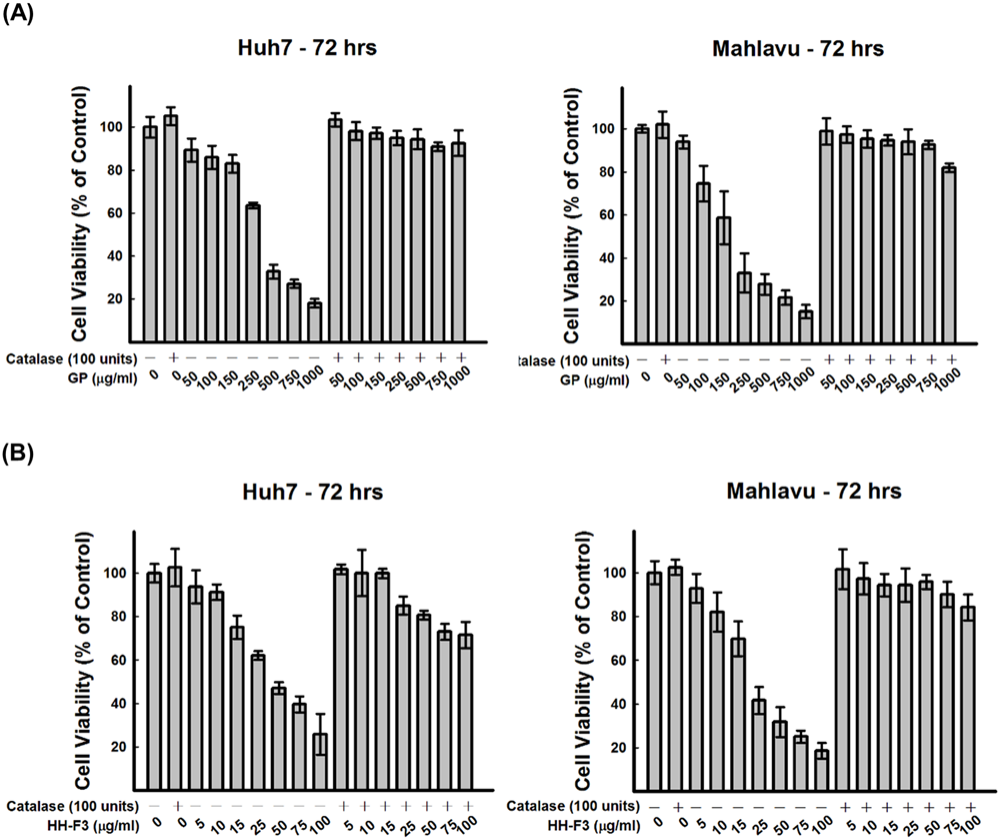
Pretreatment with catalase blocks 30% DMSO GP extracts- or HH-F3-induced cytotoxicity in HCC cells. Huh7 and Mahlavu cells were pretreated with catalase (100 U/ml) for 30 min and exposed to (A) 30% DMSO GP extracts or (B) HH-F3 for 72 hrs. Cell viability was evaluated with the MTT assay. The results were expressed as the optical density percentage relative to the control (n = 3).

Several reports have shown that ROS cause the collapse of mitochondria and that the loss of ΔΨ can trigger a further burst of ROS production [28, 29]. Superoxide anions (O_2_^-^), hydrogen peroxide and hydroxyl radicals, all of which are oxygen derivatives, are ROS. We tested whether treating the HCC cells with the HH-F3 fraction would result in changes in the production of ROS. The intracellular generation of superoxide was assessed by hydroethidine fluorescence, and the level of intracellular peroxide was determined using DCFH diacetate fluorescence. The production of superoxide (S3B Fig) and intracellular peroxide in HCC cells treated with HH-F3 (Fig 3C–3D and S3C Fig) was increased in a concentration-dependent manner. The free radical scavenger catalase (CAT) was used in the present study. As shown in Fig 4, CAT protected the HCC cells against GP/HH-F3-induced cytotoxicity.

### The HH-F3 fraction leads to cell death via PTEN-AKT-mediated apoptosis in HCC cell lines

**Fig 5:**
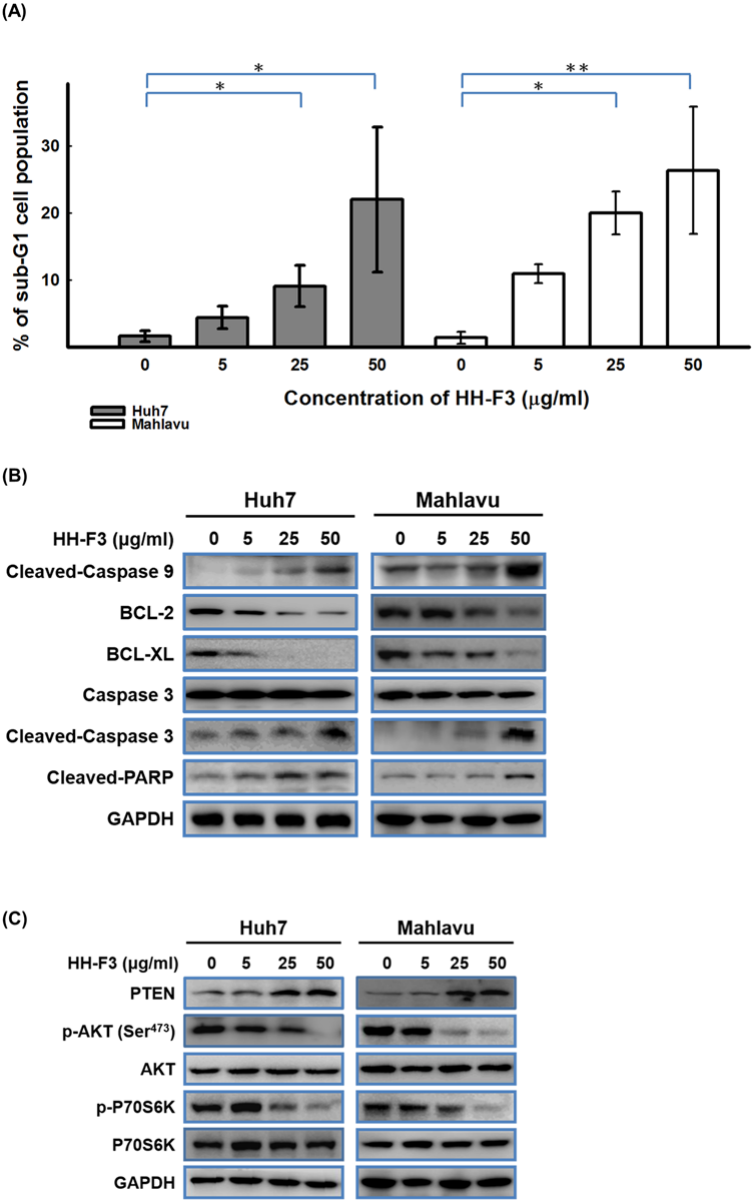
HH-F3 induces apoptosis in HCC cells. Huh7 and Mahlavu cells were treated with 5, 25 and 50 μg/ml of HH-F3 for 48 hrs. The cells were stained with propidium iodide (PI) and the DNA content of the cells was analyzed by flow cytometry to identify the distribution of sub-G1 cells in the cell cycle (*P < 0.05, **P < 0.005). (B) The cell lysates treated with the HH-F3 fraction were subjected to immunoblot analysis for anti-cleaved caspase9, anti-cleaved caspase-3 and cleaved PARP. The expression levels of cleaved caspase-3, caspase-9 and PARP were increased, indicating that the cells had undergone apoptosis. HH-F3 treatment also resulted in the down-regulation of the expression of BCL2 and BCL-XL. (C) The expression of p-AKT-Ser^473^ and p-p70S6K was down-regulated and the expression of PTEN was up-regulated by HH-F3 in a concentration-dependent manner, as shown by the results of the immunoblot analysis with anti-p70S6K, anti-p-p70S6K, anti-p-AKT-Ser^473^, anti-AKT and anti-PTEN antibodies.

We next analyzed the effects of the HH-F3 fraction on the cell cycle profiles of HCC cells using propidium iodide (PI) staining. Huh7 and Mahlavu cells were treated with 5, 25 and 50 μg/ml of HH-F3 for 48 hrs. As shown in S2 Table, the HH-F3 fraction disrupted the progression of Huh7 and Mahlavu cells through the cell cycle. After 48 hrs of treatment with 50 μg/ml of HH-F3, only 22% of the Huh7 cells and 26% of the Mahlavu cells had entered the sub-G1 phase. More Mahlavu cells treated with HH-F3 than Huh7 cells treated with HH-F3 entered the sub-G1 phase, which is consistent with the cytotoxic effects of HH-F3 demonstrated earlier (Fig 5A). The increase in the number of cells in the sub-G1 phase indicated that the HH-F3 fraction induced apoptosis or necrosis of Huh7 and Mahlavu cells. Because reactive oxygen species (ROS) and mitochondria play an important role in inducing apoptosis under physiological and pathological conditions, we next investigated whether HH-F3 triggers apoptosis via the extrinsic or intrinsic pathway. We further examined the ability of the HH-F3 fraction to induce apoptotic cell death in Huh7 and Mahlavu cells. The levels of cleaved caspase-3, caspase-9 and PARP proteins were increased in a concentration-dependent manner (Fig 5B). HH-F3 treatment also resulted in the down-regulation of the anti-apoptotic proteins BCL2 and BCL-XL (Fig 5B and S5A Fig). These data suggest that the HH-F3 fraction triggered the intrinsic pathway, causing caspase-dependent apoptotic cell death via the production of ROS.

Certain cell proliferation pathways such as the AKT pathway are related to the inhibition of apoptosis and abnormalities in HCC cells. Because HH-F3 was cytotoxic to HCC cells, we investigated whether HH-F3 affected cell proliferation pathways in HCC cells. Huh7 and Mahlavu cells were treated with 5, 25 and 50 μg/ml of HH-F3 for 48 hrs. Ser^473^-phosphorylated AKT levels were down-regulated by the HH-F3 fraction, whereas total AKT protein levels were not altered (Fig 5C and S5B Fig). Interestingly, HH-F3 increased the protein levels of phosphatase and tensin homolog (PTEN), a negative regulator of PI3K/AKT-dependent signaling. These results suggest that the pro-apoptotic effects of HH-F3 are mediated by the inhibition of the AKT signaling pathway. We also found that the 30% DMSO GP extracts and HH-F3 similarly affected the AKT pathway (S4 Fig) in HCC cell lines.

### GP extracts, HH-F3 and sorafenib have synergistic effects

**Fig 6:**
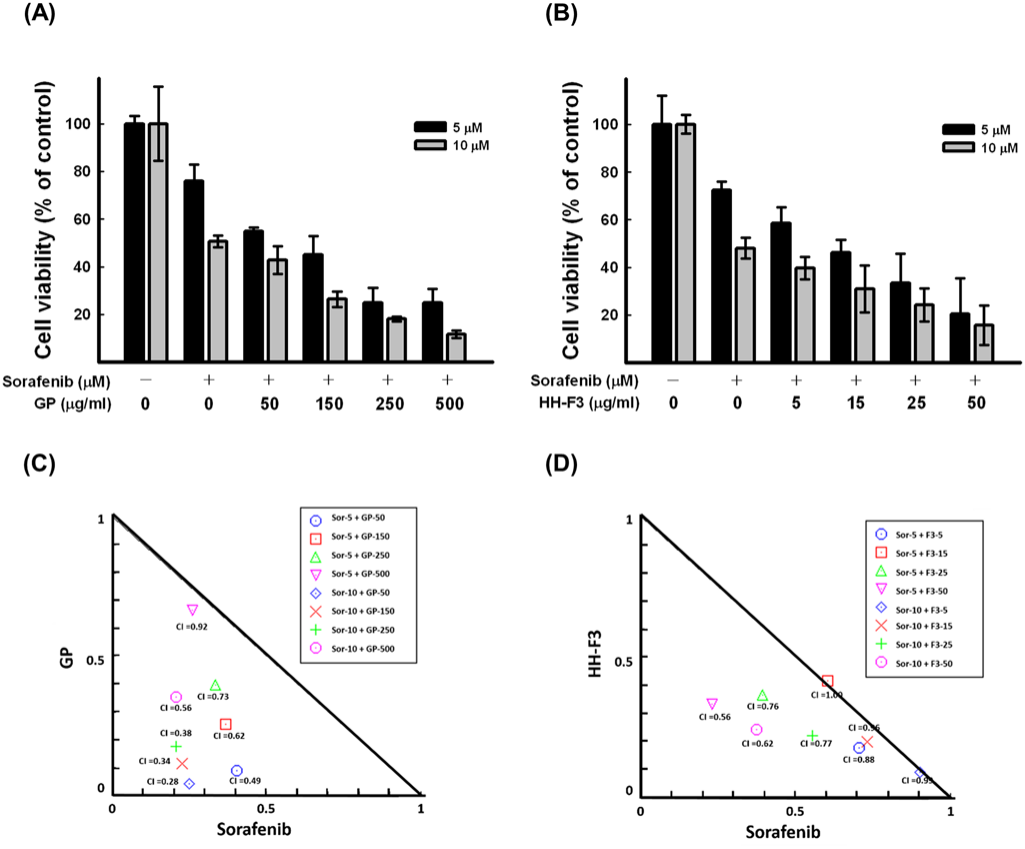
Synergistic effect of the combination of GP extracts/HH-F3 and sorafenib on the viability of Huh7 cells. (A, B) Huh7 cells were treated with various concentrations of 30% DMSO GP extracts/ HH-F3 in the presence or absence of sorafenib (5 μM and 10 μM). Cell viability was determined with the SRB assay. Data are expressed as the mean ± standard deviation (SD) of three independent experiments. (C, D) Isobologram analysis demonstrates the synergistic interaction between sorafenib and various concentrations of GP/HH-F3 in Huh7 cells at 72 hrs.

Sorafenib is an FDA-approved agent for the treatment of HCC; however, the extent to which the drug induces tumor shrinkage is minor. We next tested the effects of combinations of various concentrations of GP or HH-F3 and sorafenib on the proliferation of Huh7 cells 72 hrs after treatment (Fig 6A–6B). We then performed combination index (CI) analysis (Fig 6C–6D) using the Chou-Talalay method to assess whether the interactions between these targeted therapies were synergistic (CI < 1), additive (CI = 1) or antagonistic (CI > 1) [30]. The results of these analyses indicate that the combination of GP and/or HH-F3 and sorafenib synergistically inhibits the proliferation of Huh7 cells.

### GP extracts increase bile excretion in cirrhotic animals

**Fig 7:**
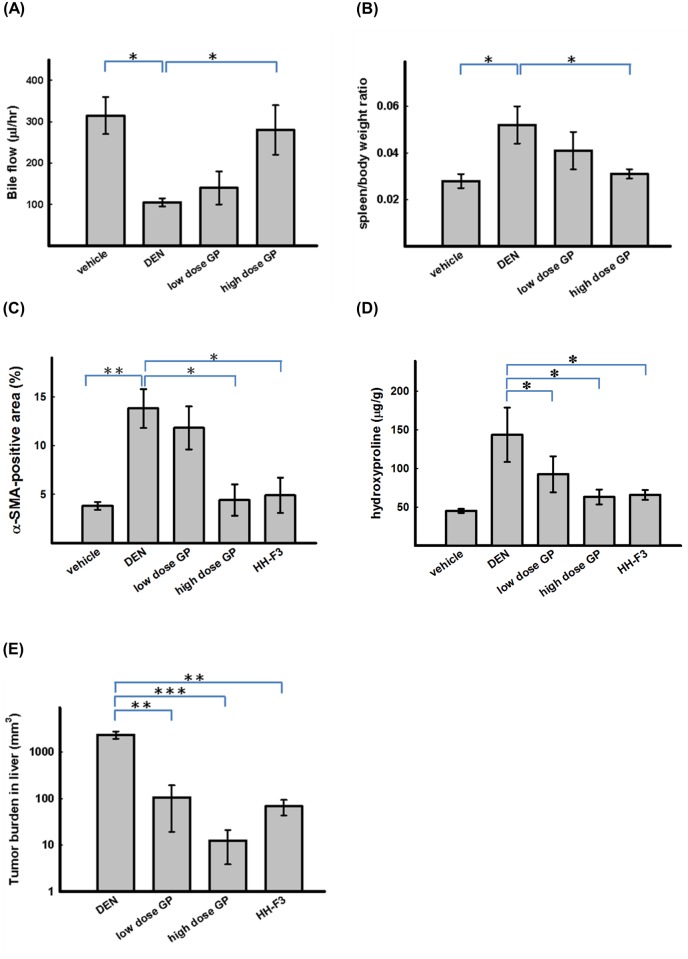
GP extracts and HH-F3 alleviate DEN-induced liver fibrosis and cancer in rats. The animals were divided into four groups and provided with tap water only (normal group) or with DEN-containing water (liver disease group). (A) Decreased bile flow in cirrhotic animals. The bile flow rate was recorded to measure liver function. *P < 0.05; ANOVA. (B) The spleen weight-to-body weight (BW) ratio of the animals in the high-dose GP treatment group was significantly lower than that of the animals in the DEN group (P < 0.05, ANOVA). (C) Treatment with GP extracts and HH-F3 significantly decreased liver hydroxyproline levels. (D) The percentages of α-SMA (+) areas were determined with a digital camera system using the 10 fields with the densest staining. *P < 0.05; **P < 0.005, ANOVA. (E) Tumor burdens were expressed as the sum of the volume of all tumor nodules. **P < 0.005, ***P < 0.001 relative to the DEN group.

We induced chronic liver disease in the experimental animals using DEN, a well-known chemical carcinogen that stimulates the hepatic inflammation-fibrosis-cancer axis. The degree of cirrhosis in these animals was evaluated by measuring bile flow rates, which reflect liver function (Fig 7A), by quantifying the spleen weight-to-body weight ratio (Fig 7B), an indicator of cirrhosis-related portal hypertension, and by analyzing the expression of α-SMA induced by DEN (Fig 7C).

Enlarged spleens in cirrhotic animals. The spleen weights and body weights (BWs) were measured and expressed as spleen weight/BW. The spleen weight-to-BW ratios were significantly higher in the animals of the DEN group than in the animals of the normal group, indicating that the splenomegaly observed in these animals was associated with cirrhosis-related portal vein hypertension. All of the data demonstrated that the severity of cirrhosis was mitigated in animals administered a high-dose of GP; indeed, these animals had significantly increased bile flow, significantly smaller spleens and significantly decreased levels of α-SMA expression.

### GP extracts and HH-F3 decrease hydroxyproline levels in cirrhotic livers

Collagen contents are increased in cirrhotic livers. Therefore, the degree of cirrhosis was assessed by measuring levels of hepatic hydroxyproline, as described in the Materials and Methods section. A significant increase in hydroxyproline levels was observed in DEN-treated animals (143 ± 30 μg/g). In contrast, DEN-treated animals administered a low or high dose of GP or HH-F3 had lower levels of hydroxyproline (98 ± 18, 70 ± 10 and 72 ± 8.2 μg/g, respectively) (Fig 7D).

### GP extracts and HH-F3 decrease tumor burden

The livers obtained from the sacrificed animals were sliced into 5-mm sections. The numbers and sizes of all visible tumor nodules with diameters larger than 3 mm were counted and measured, respectively. The tumor burden was expressed as the sum of the volume of all tumor nodules. Visible tumors were observed in DEN-treated animals (tumor burden 2350 ± 905 mm^3^). In contrast, the hepatic tumor burden of DEN-treated animals administered a low or high dose of GP or HH-F3 was reduced to 110 ± 105, 23 ± 31 and 86 ± 12 mm^3^, respectively (Fig 7E). We observed granulation on the surface of the liver and an uneven boundary with multiple hepatic tumors in the GP/HH-F3-treated animals; the numbers of visible tumors and the degree of unevenness on the surfaces of the livers were decreased and improved, respectively, in the animals treated with a low or high dose of GP or HH-F3.

## Discussion

GP is a commonly used herbal medicine in Taiwan. GP extracts have been shown to have anti-fibrotic and anti-inflammatory effects *in vivo* and *in vitro*. In this study, we obtained the HH-F3 fraction as the active component for anti-cancer activity from the 30% DMSO GP extracts. The broadened aromatic signals in ^1^H and ^13^C NMR spectra identified the major components of this fraction as polyphenolic compounds. The anti-cancer effects of proanthocyanidins have previously been described [31–33]; however, the mechanisms by which they exert these anti-cancer effects at the cellular, subcellular and genetic levels remain unclear. Here, we showed that the proanthocyanidin-rich HH-F3 fraction suppresses AURKA and AURKB protein expression (Fig 1) and disrupts the ability to progress in the cell cycle and the mitotic integrity of HCC.

Use of herbal medicine and nature products in the treatment of liver cancer has a long tradition. Many Chinese herbal medicine have anti-HCC activity in previous reports, for example, the extracts prepared from the roots of *Paeonia lactiflora* and *Astragalus membranaceus* (PAE) induces apoptosis and inhibits the proliferation, migration and invasion of human hepatoma cell lines [34]; Sho-saiko-to (TJ-9) prevents the development of HCC in patients with cirrhosis, particularly in patients with negative HBs antigen [35]; Juzen-taiho-to (TJ-48) inhibits tumors growth and improves intrahepatic recurrence-free survival after surgical treatment of HCC [36]. Natural products such as plant polyphenolic compounds have been extensively studied for their chemopreventive and chemotherapeutic potential [37, 38]. These products can be used as novel naturally derived anti-cancer therapeutics [39]; they can also be used for nutraceutical therapy. Various compounds have been examined under different circumstances. Recently, a significant amount of research has focused on the identification of phytochemicals—both nutrient and non-nutrient—in fruits and vegetables. Many of these phytochemicals exert beneficial effects, and studies have focused on understanding the mechanisms by which they inhibit cellular injury and degeneration. In the last decade, there has been great interest in bioflavonoid and phytochemical research; in particular, pycnogenol, quercetin, resveratrol, curcumin, extracts of green and black tea, soy proteins, isoflavones and proanthocyanidin-rich grape seed extracts (GSPE) appear to be promising agents for preventing disease [38].

Our previous study indicated that the overexpression of FLJ10540 contributed to cellular transformation via the activation of the PI3K/AKT pathway [22]. Another study showed that PTEN was able to regulate AURKA expression levels via the PI3K/AKT/GSK3β pathway [40]. The silencing of AURKA by shRNAs or small molecular inhibitors induced apoptosis and inhibited the growth of cancer cells and tumors in various cancers [41–43]. The PI3K/AKT pathway plays a critical role in carcinogenesis. The activation of this pathway may result from increased signaling due to the overexpression of ligands (i.e., EGF, IGF1 and IGF2) or from mutations in *PI3KCA* or *PTEN*. Certain cancer patients can have a simultaneous activation of AKT. It is known that AKT activity can be positively regulated by PI3K and negatively regulated by PTEN. Many studies have demonstrated that PI3K can maintain cell survival and prevent apoptosis [44, 45]. The current study shows that the HH-F3 fraction has anti-cancer effects, as it is able to inhibit the PI3K/AKT pathway by up-regulating the expression of PTEN and selectively activating apoptotic pathways (Fig 5). And then we use microarray to analysis HCC treated with HH-F3. Our microarray data indicate that HCC cells treated with HH-F3 did not affect *PTEN* gene expression levels, suggesting that HH-F3 mediated up regulation PTEN expression is at the protein level rather than at the transcriptional level.

In our microarray analysis data also indicated that the expression of certain apoptotic genes, such as *BCL2L11*, *BIK*, *DDIT4*, *FAS* and *PMAIP1*, were up-regulated (data not shown). Apoptosis can be initiated through an extrinsic pathway, also referred to as the death receptor pathway, or an intrinsic pathway, also referred to as the mitochondrial pathway. The extrinsic pathway is associated with the activation of caspase-8, and the intrinsic pathway is associated with the activation of caspase-9. These two pathways converge on the activation of caspase-3 and trigger apoptosis [46]. This study showed that HH-F3 induced apoptosis in HCC cell lines through the intrinsic pathway by activating caspase-9. The intrinsic pathway is a signal transduction pathway involving the mitochondria and the Bcl-2 protein family. Here, we found that the mitochondrial membrane potential (ΔΨ) was significantly decreased in Huh7 and Mahlavu cells treated with the HH-F3 fraction (Fig 3A). It has been demonstrated that cells with ruptured mitochondria, which experience a loss of the electrochemical gradient across the inner mitochondrial membrane, are at increased risk of cell death due to apoptotic processes or nonapoptotic mechanisms that resemble necrosis [47]. Initially, we speculated that the HH-F3 fraction might induce apoptosis in hepatoma cells via the intrinsic pathway. Excessive ROS production causes oxidative damage to mitochondrial proteins, membranes and DNA, thus impairing the ability of mitochondria to synthesize ATP [48]. Together, HH-F3 increased ROS production in HCC cell lines (Fig 3C and 3D) and triggered a mitochondria-mediated apoptotic pathway (S3 Fig).

Recent evidence suggests that cross-talk between the Ras/Raf/MAPK and PI3K/AKT pathways occurs in a number of cancers [49]. Moreover, the sorafenib-mediated inhibition of DEP-1 phosphatase activity has been shown to result in in the phosphorylation of c-Met at Tyr^1349^, which leads to the activation of the PI3K-AKT pathway [50]. Therefore, the combination of sorafenib and GP/HH-F3 may target both facets of this cross-talk (Ras/Raf and PI3K/AKT), thus producing the observed synergistic effects (Fig 6).

Human HCC is a unique disease, as it is often complicated by cirrhosis. Rodent models for understanding the progression from fibrosis to HCC have been developed. Carbon tetrachloride (CCl_4_), DMN and DEN are the most commonly used hepatotoxic agents; these agents produce bridge fibrosis that develops initially in the pericentral areas and subsequently between the central and portal areas, eventually leading to HCC and cirrhosis [51, 52]. Our study clearly indicated that the administration of the GP extracts or the HH-F3 fraction decreased hepatic collagen levels and inhibited tumor growth in a rat model with diethylnitrosamine (DEN)-induced liver fibrosis and cancer (Fig 7).

In conclusion, our findings provide evidence that GP extracts and the active HH-F3 fraction could be used as anti-cancer agents that suppress oncogenic protein expression in human HCC cell lines and mitigate DEN-induced liver fibrosis and cancer in rats. Sorafenib acted synergistically with GP/HH-F3 in *in vitro* HCC cell culture experiments. These observations suggest that the compounds present in GP have potential therapeutic effects and can slow the progression of disease in patients with HCC and liver cirrhosis.

## Supporting Information

S1 Fig Regulation of AURK protein expression in Huh7cells by GP extractsprepared with different solvents. (A) Huh7 cells were treated with 500 μg/ml GP extracts (prepared with different solvents, including H2O, acetone, methanol, 100% ethanol, 70% ethanol, 50% ethanol, 100% DMSO or 30% DMSO) for 48 hrs. (B) The protein expression of AURKA was inhibited by the 30% DMSO GP extracts. Huh7 and HepG2 cells were treatedwith 0, 375, 750 and 1,500 μg/ml of 30% DMSO GP extracts for 24 hrs. Cell lysates were subjected to immunoblot analysis with anti-AURKA, anti-AURKB, and anti-FLJ10540 antibodies.
(PDF)

S2 Fig Preparation of GP extracts and the HH-F3 fraction. (A) Flowchart for preparation of GP extracts and the HH-F3 fraction (B) HPLC fingerprint of HH-F3. The HH-F3 fraction was analyzed by high-performance liquid chromatography (HPLC) with a UV detector and a normal-phase HPLC column. (C) Proposed chemical structure of the major component in the HH-F3a fraction. Prodelphinidin repeating units connected with (4→8)-linkages.
(PDF)

S3 Fig The HH-F3 fraction decreases mitochondrial membrane potential and increases ROS generation in HCC cell lines. (A) The mitochondrial membrane potential (ΔΨ) in Huh7 and Mahlavu cells was analyzed using the JC-1 fluorescence probe. The ΔΨ was lower in the cells treated with 5, 10, 15, 25 and 50 μg/ml of the HH-F3 fraction for 24 hrs than in the control HCCcells (treated with 0.1% DMSO) (n = 5). Relative ratio of red to green fluorescence = ΔΨ. (B) Intracellular superoxide (O2-) levels were measured by hydroethidine (HE) staining. Superoxide levelsincreased 24 hrs after treatment with HH-F3 (n = 3).(C) Intracellular peroxide levels were measured by DCFH fluorescence. Peroxide levels increased 24 hrs after treatment with HH-F3 (n = 5).*P < 0.05, **P < 0.005.
(PDF)

S4 Fig Modulation of the expression of AURKA, FLJ10540, PTEN, AKT, PI3K, p70S6K and cleaved PARP by 30% DMSO GP extracts in HCC cell lines. The cell lysates treated with 30% DMSO GP extractswere subjected to immunoblot analysis for anti-cleaved PARP, anti-FLJ10540, anti-AURKA, anti-p70S6K, anti-AKT-Ser473, anti-AKT and anti-PTEN antibodies. The expression levels of cleaved PARP were increased, indicating that the cells had undergone apoptosis. The expression of AURKA, FLJ10540 and AKT-Ser473 was down-regulated, whereas that of PTEN was up-regulated in a concentration-dependent manner.
(PDF)

S5 Fig HH-F3 induce apoptosis via regulation of PTEN/AKT pathway. (A) The time-dependent effect of HH-F3 treatment (50 μg/ml) on the expression of apoptosis-related proteins (BCL-2, BCL-XL, cleaved caspase9, cleaved caspase-3 and cleaved PARP) as assayed by western blot. (B) Time-dependent western blot analysis of PTEN/AKT pathway after HH-F3 treatment (50 μg/ml).
(PDF)

S1 Table Physicochemical properties of the polymeric proanthocyanidin from *P*. *paraguayense*, *R*. *rosea*, and two different parts of *Vitis vinifera*. (PDF)

S2 Table Increase in the number of HH-F3-treated cells in the sub-G1 phase. Huh7 and Mahlavu cells were treated with 5, 25 and 50 μg/ml of HH-F3 for 48 hrs. The cells were stained with propidium iodide (PI), and the DNA content of the cells was analyzed by flow cytometry. The increased number of cells in the sub-G1 phase indicated that HH-F3 induced apoptosis or necrosis in Huh7 and Mahlavu cells (*P < 0.05, **P < 0.001).
(PDF)

S3 Table Effect of 30% DMSO GP extracts and HH-F3 in various cancer cell lines. (PDF)
