# Supplementary material for: Evaluation of the Medicinal Herb Graptopetalum paraguayense as a Treatment for Liver Cancer
Source: PLoS One. 2015 Apr 7;10(4):e0121298. doi: 10.1371/journal.pone.0121298 (PMC4388720; doi:10.1371/journal.pone.0121298)
Supplement: S1 Fig — (A) Huh7 cells were treated with 500 μg/ml GP extracts (prepared with different solvents, including H2O, acetone, methanol, 100% ethanol, 70% ethanol, 50% ethanol, 100% DMSO or 30% DMSO) for 48 hrs. (B) The protein expression of AURKA was inhibited by the 30% DMSO GP extracts. Huh7 and HepG2 cells were treatedwith 0, 375, 750 and 1,500 μg/ml of 30% DMSO GP extracts for 24 hrs. Cell lysates were subjected to immunoblot analysis with anti-AURKA, anti-AURKB, and anti-FLJ10540 antibodies. (PDF) [file pone.0121298.s001.pdf]

(A)

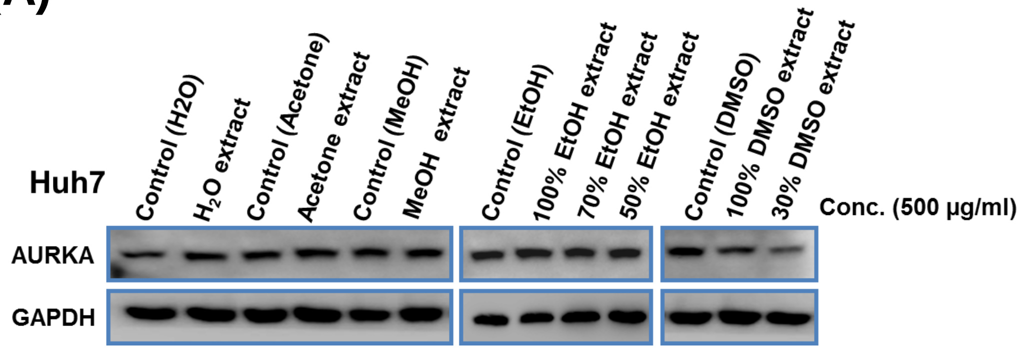

(B)

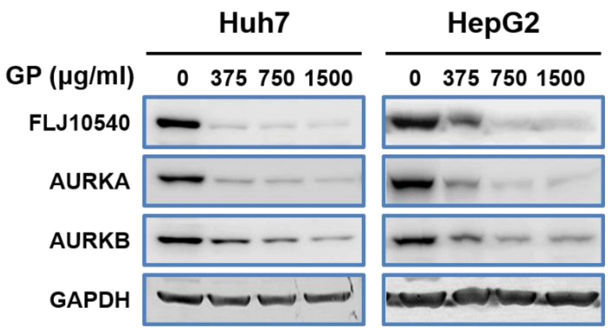

S1 Fig. Regulation of AURK protein expression in Huh7 cells by GP extracts prepared with different solvents.

(A) Huh7 cells were treated with 500 μg/ml GP extracts (prepared with different solvents, including H_2O, acetone, methanol, 100% ethanol, 70% ethanol, 50% ethanol, 100% DMSO or 30% DMSO) for 48 hrs. (B) The protein expression of AURKA was inhibited by the 30% DMSO GP extracts. Huh7 and HepG2 cells were treated with 0, 375, 750 and 1,500 μg/ml of 30% DMSO GP extracts for 24 hrs. Cell lysates were subjected to immunoblot analysis with anti-AURKA, anti-AURKB, and anti-FLJ10540 antibodies.
