# Supplementary material for: Evaluation of the Medicinal Herb Graptopetalum paraguayense as a Treatment for Liver Cancer
Source: PLoS One. 2015 Apr 7;10(4):e0121298. doi: 10.1371/journal.pone.0121298 (PMC4388720; doi:10.1371/journal.pone.0121298)
Supplement: S2 Fig — (A) Flowchart for preparation of GP extracts and the HH-F3 fraction (B) HPLC fingerprint of HH-F3. The HH-F3 fraction was analyzed by high-performance liquid chromatography (HPLC) with a UV detector and a normal-phase HPLC column. (C) Proposed chemical structure of the major component in the HH-F3a fraction. Prodelphinidin repeating units connected with (4→8)-linkages. (PDF) [file pone.0121298.s002.pdf]

(A)

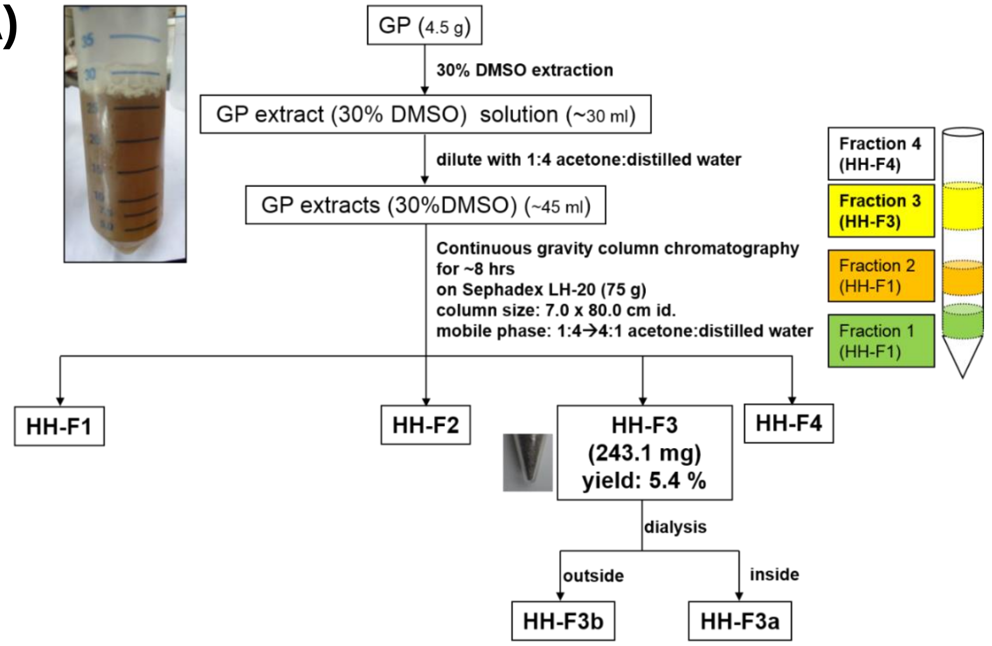
GP (4.5 g)
30% DMSO extraction
GP extract (30% DMSO) solution (~30 ml)
dilute with 1:4 acetone:distilled water
GP extracts (30%DMSO) (~45 ml)
Continuous gravity column chromatography
for ~8 hrs
on Sephadex LH-20 (75 g)
column size: 7.0 x 80.0 cm id.
mobile phase: 1:4→4:1 acetone:distilled water
Fraction 4 (HH-F4)
Fraction 3 (HH-F3)
Fraction 2 (HH-F1)
Fraction 1 (HH-F1)
HH-F1
HH-F2
HH-F3 (243.1 mg) yield: 5.4 %
HH-F4
dialysis
outside
inside
HH-F3b
HH-F3a

(B)

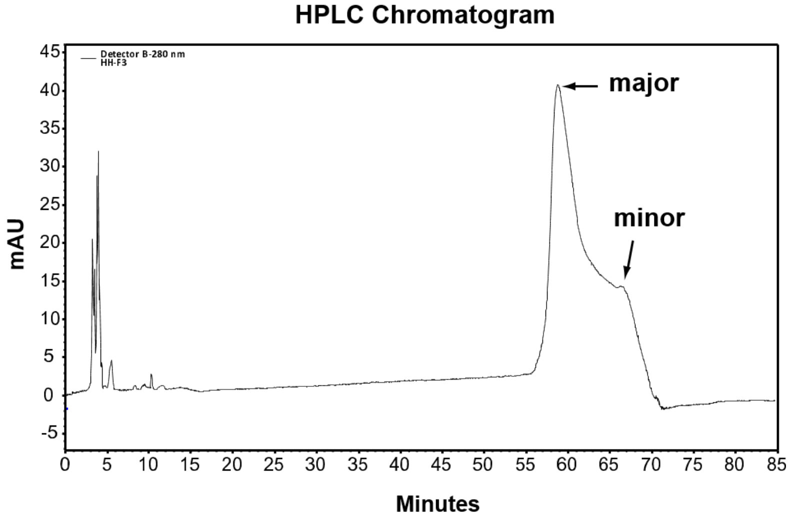
HPLC Chromatogram
Detector B-280 nm
HH-F3
major
minor
mAU
45
40
35
30
25
20
15
10
5
0
-5
0 5 10 15 20 25 30 35 40 45 50 55 60 65 70 75 80 85
Minutes

(C)

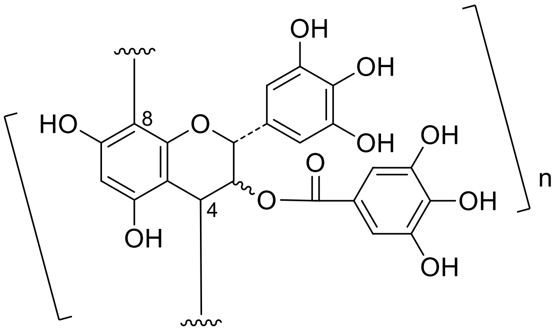
OH
OH
OH
HO
8
O
O
OH
OH
OH
OH
4
O
n

S2 Fig. Preparation of GP extracts and the HH-F3 fraction.

(A) Flowchart for preparation of GP extracts and the HH-F3 fraction (B) HPLC fingerprint of HH-F3. The HH-F3 fraction was analyzed by high-performance liquid chromatography (HPLC) with a UV detector and a normal-phase HPLC column. (C) Proposed chemical structure of the major component in the HH-F3a fraction. Prodelphinidin repeating units connected with (4$\rightarrow$8)-linkages.
