# Supplementary material for: Evaluation of the Medicinal Herb Graptopetalum paraguayense as a Treatment for Liver Cancer
Source: PLoS One. 2015 Apr 7;10(4):e0121298. doi: 10.1371/journal.pone.0121298 (PMC4388720; doi:10.1371/journal.pone.0121298)
Supplement: S3 Fig — (A) The mitochondrial membrane potential (ΔΨ) in Huh7 and Mahlavu cells was analyzed using the JC-1 fluorescence probe. The ΔΨ was lower in the cells treated with 5, 10, 15, 25 and 50 μg/ml of the HH-F3 fraction for 24 hrs than in the control HCCcells (treated with 0.1% DMSO) (n = 5). Relative ratio of red to green fluorescence = ΔΨ. (B) Intracellular superoxide (O2-) levels were measured by hydroethidine (HE) staining. Superoxide levelsincreased 24 hrs after treatment with HH-F3 (n = 3).(C) Intracellular peroxide levels were measured by DCFH fluorescence. Peroxide levels increased 24 hrs after treatment with HH-F3 (n = 5).*P < 0.05, **P < 0.005. (PDF) [file pone.0121298.s003.pdf]

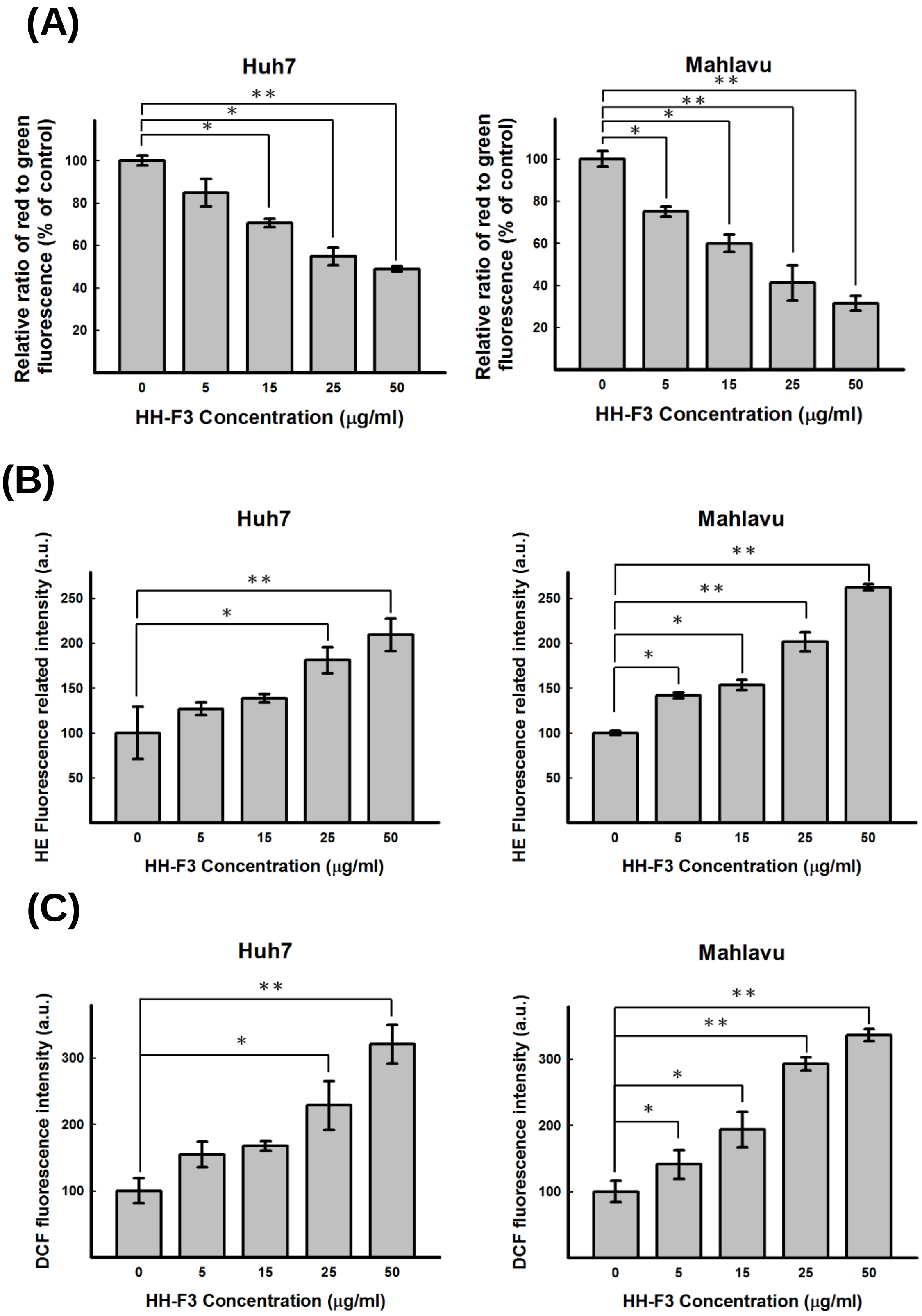
(A)
Huh7
Relative ratio of red to green fluorescence (% of control)
HH-F3 Concentration (μg/ml)
Mahlavu
Relative ratio of red to green fluorescence (% of control)
HH-F3 Concentration (μg/ml)
(B)
Huh7
HE Fluorescence related intensity (a.u.)
HH-F3 Concentration (μg/ml)
Mahlavu
HE Fluorescence related intensity (a.u.)
HH-F3 Concentration (μg/ml)
(C)
Huh7
DCF fluorescence intensity (a.u.)
HH-F3 Concentration (μg/ml)
Mahlavu
DCF fluorescence intensity (a.u.)
HH-F3 Concentration (μg/ml)

S3 Fig. The HH-F3 fraction decreases mitochondrial membrane potential and increases ROS generation in HCC cell lines.

(A) The mitochondrial membrane potential ($\Delta\Psi$) in Huh7 and Mahlavu cells was analyzed using the JC-1 fluorescence probe. The $\Delta\Psi$ was lower in the cells treated with 5, 10, 15, 25 and 50 μg/ml of the HH-F3 fraction for 24 hrs than in the control HCC cells (treated with 0.1% DMSO) (n =5). Relative ratio of red to green fluorescence = $\Delta\Psi$. (B) Intracellular superoxide (O_2^-) levels were measured by hydroethidine (HE) staining. Superoxide levels increased 24 hrs after treatment with HH-F3 (n =3). (C) Intracellular peroxide levels were measured by DCFH fluorescence. Peroxide levels increased 24 hrs after treatment with HH-F3 (n =5). *$P < 0.05$, **$P < 0.005$
