# Supplementary material for: Evaluation of the Medicinal Herb Graptopetalum paraguayense as a Treatment for Liver Cancer
Source: PLoS One. 2015 Apr 7;10(4):e0121298. doi: 10.1371/journal.pone.0121298 (PMC4388720; doi:10.1371/journal.pone.0121298)
Supplement: S4 Fig — The cell lysates treated with 30% DMSO GP extractswere subjected to immunoblot analysis for anti-cleaved PARP, anti-FLJ10540, anti-AURKA, anti-p70S6K, anti-AKT-Ser473, anti-AKT and anti-PTEN antibodies. The expression levels of cleaved PARP were increased, indicating that the cells had undergone apoptosis. The expression of AURKA, FLJ10540 and AKT-Ser473 was down-regulated, whereas that of PTEN was up-regulated in a concentration-dependent manner. (PDF) [file pone.0121298.s004.pdf]

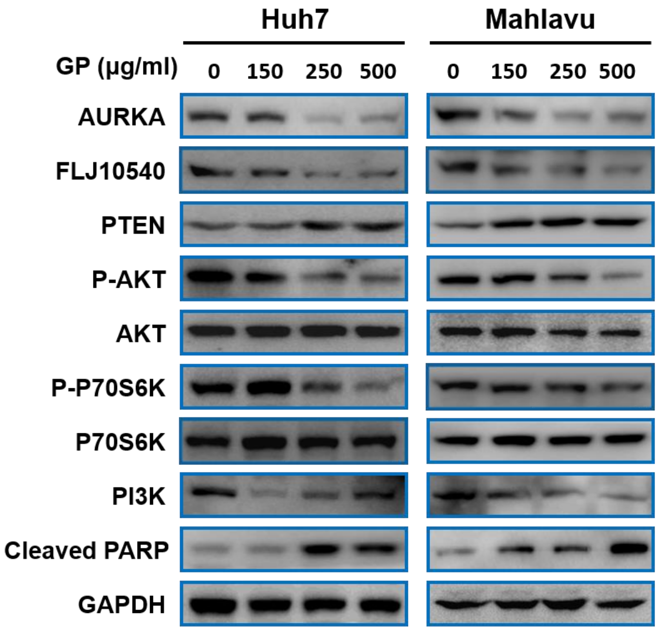

S4 Fig. Modulation of the expression of AURKA, FLJ10540, PTEN, AKT, PI3K, p70S6K and cleaved PARP by 30% DMSO GP extracts in HCC cell lines.

The cell lysates treated with 30% DMSO GP extracts were subjected to immunoblot analysis for anti-cleaved PARP, anti-FLJ10540, anti-AURKA, anti-p70S6K, anti-AKT-Ser^{473}, anti-AKT and anti-PTEN antibodies. The expression levels of cleaved PARP were increased, indicating that the cells had undergone apoptosis. The expression of AURKA, FLJ10540 and AKT-Ser^{473} was down-regulated, whereas that of PTEN was up-regulated in a concentration-dependent manner.
