# Supplementary material for: Evaluation of the Medicinal Herb Graptopetalum paraguayense as a Treatment for Liver Cancer
Source: PLoS One. 2015 Apr 7;10(4):e0121298. doi: 10.1371/journal.pone.0121298 (PMC4388720; doi:10.1371/journal.pone.0121298)
Supplement: S5 Fig — (A) The time-dependent effect of HH-F3 treatment (50 μg/ml) on the expression of apoptosis-related proteins (BCL-2, BCL-XL, cleaved caspase9, cleaved caspase-3 and cleaved PARP) as assayed by western blot. (B) Time-dependent western blot analysis of PTEN/AKT pathway after HH-F3 treatment (50 μg/ml). (PDF) [file pone.0121298.s005.pdf]

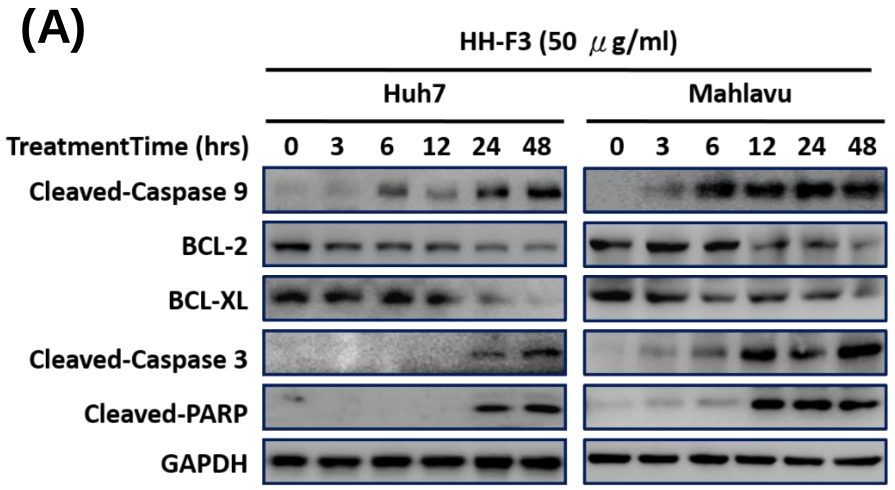

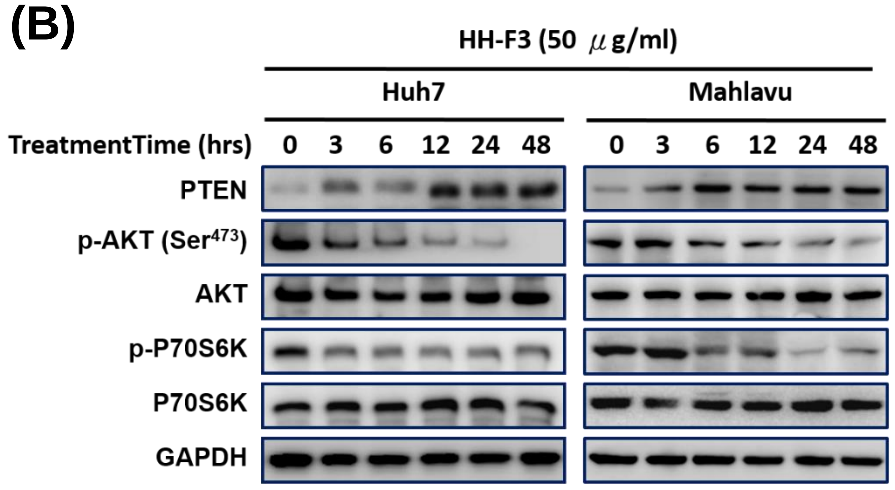

S5 Fig. HH-F3 induce apoptosis via regulation of PTEN/AKT pathway

(A) The time-dependent effect of HH-F3 treatment (50 μg/ml) on the expression of apoptosis-related proteins (BCL-2, BCL-XL, cleaved caspase9, cleaved caspase-3 and cleaved PARP) as assayed by western blot. (B) Time-dependent western blot analysis of PTEN/AKT pathway after HH-F3 treatment (50 μg/ml).
