# Supplementary material for: Evaluation of the Medicinal Herb Graptopetalum paraguayense as a Treatment for Liver Cancer
Source: PLoS One. 2015 Apr 7;10(4):e0121298. doi: 10.1371/journal.pone.0121298 (PMC4388720; doi:10.1371/journal.pone.0121298)
Supplement: S1 Table — (PDF) [file pone.0121298.s006.pdf]

S1 Table. Physiochemical properties of the polymeric proanthocyanidin from *P. paraguayense, R. rosea* and two different parts of *Vitis vinifera*

Source	Part	Preparation	3-*O*-galloyl (%)	mMW (kD)
P. paraguayense	leaf	Method I<br>1. Extraction by DMSO-H_2O (3:7)<br>2. Sephadex LH-20 chromatography<br>Method II<br>1. Extraction by DMSO-H_2O (3:7)<br>2. Dialysis	> 95	18
Rhodiola rosea	leaf	Extraction by acetone-H_2O (7:3)	> 95	6.0
Vitis vinifera[1]	seed	Extraction by acetone-H_2O (6:4)	20.4	2.6
Vitis vinifera	skin	Extraction by acetone-H_2O (6:4)	2.3	10.4

[1] The European grapevine native to the Mediterranean region and central Asia
