# Supplementary material for: Evaluation of the Medicinal Herb Graptopetalum paraguayense as a Treatment for Liver Cancer
Source: PLoS One. 2015 Apr 7;10(4):e0121298. doi: 10.1371/journal.pone.0121298 (PMC4388720; doi:10.1371/journal.pone.0121298)
Supplement: S2 Table — Huh7 and Mahlavu cells were treated with 5, 25 and 50 μg/ml of HH-F3 for 48 hrs. The cells were stained with propidium iodide (PI), and the DNA content of the cells was analyzed by flow cytometry. The increased number of cells in the sub-G1 phase indicated that HH-F3 induced apoptosis or necrosis in Huh7 and Mahlavu cells (*P < 0.05, **P < 0.001). (PDF) [file pone.0121298.s007.pdf]

S2 Table. Increase in the number of HH-F3-treated cells in the sub-G1 phase.

Huh7 and Mahlavu cells were treated with 5, 25 and 50 μg/ml of HH-F3 for 48 hrs. The cells were stained with propidium iodide (PI), and the DNA content of the cells was analyzed by flow cytometry. The increased number of cells in the sub-G1 phase indicated that HH-F3 induced apoptosis or necrosis in Huh7 and Mahlavu cells (*P < 0.05, **P < 0.001).

S2 Table.

Huh7

Stage of cell cycle	Control	HH-F3 (5μg/ml)	HH-F3 (25μg/ml)	HH-F3 (50μg/ml)
Sub-G1	1.8±0.8	4.5±1.7	9.1±3.1*	22.0±10.8*
G0-G1	63.8±4.0	63.7±3.9	64.6±5.0	57.0±7.5
S	12.9±1.3	12.6±2.4	10.8±2.1	9.2±2.7
G2-M	20.6±3.1	18.6±3.9	15.3±5.4	11.7±4.3
>4N	0.9±0.4	0.6±0.2	0.2±0.1	0.1±0.1

Mahlavu

Stage of cell cycle	Control	HH-F3 (5μg/ml)	HH-F3 (25μg/ml)	HH-F3 (50μg/ml)
Sub-G1	1.6±0.9	11.3±1.4	20.0±3.2*	26.6±9.5**
G0-G1	57.5±3.0	50.8±4.6	49.4±10.3	42.3±10.0
S	16.4±5.8	16.5±6.5	16.3±8.1	15.1±7.1
G2-M	23.4±6.5	20.7±9.7	13.5±6.6	15.3±6.5
>4N	1.1±0.6	0.7±0.1	0.8±0.1	0.7±0.2
