# Supplementary material for: Evaluation of the Medicinal Herb Graptopetalum paraguayense as a Treatment for Liver Cancer
Source: PLoS One. 2015 Apr 7;10(4):e0121298. doi: 10.1371/journal.pone.0121298 (PMC4388720; doi:10.1371/journal.pone.0121298)
Supplement: S3 Table — (PDF) [file pone.0121298.s008.pdf]

S3 Table. Effect of 30% DMSO GP extracts and HH-F3 in various cancer cell lines

Type of Cancer	Cell Line	IC_{50} (μg/ml)	
		30% DMSO GP extracts	HH-F3
Liver cancer	HepG2	750± 41.62	75± 6.19
	HeP3B	500± 22.85	50± 11.71
	Huh7	500± 54.28	50 ± 8.13
	Mahlavu	250± 48.85	37.5 ± 5.56
	PLC5	1000 ± 51.32	100 ± 11.38
Lung cancer	A549	>1000	>100
	CL1-0	>1000	>100
	CL1-3	650± 68.68	75± 6.68
	CL1-5	500± 72.57	65± 13.18
	H1975	>1000	>100
	H1299	>1000	>100
Breast cancer	MCF7	1000± 11.01	100± 19.34
Glioblastoma multiforme	GBM8401	>1000	>100
	U87	>1000	>100
Cervical cancer	HeLa	>1000	>100
Nasopharyngeal carcinoma	TW01	>1000	>100
	TW03	>1000	>100
	TW04	>1000	>100
Prostate cancer	PC3	>1000	>100
Colon cancer	HT29	>1000	>100
